# Distributed time-varying out formation-containment tracking of multi-UAV systems based on finite-time event-triggered control

**DOI:** 10.1038/s41598-022-24083-y

**Published:** 2022-11-24

**Authors:** Xin Cai, Xiaozhou Zhu, Wen Yao

**Affiliations:** Defense Innovation Institute, Chinese Academy of Military Science, Beijing, 100000 China

**Keywords:** Aerospace engineering, Electrical and electronic engineering, Applied mathematics, Computer science, Information technology

## Abstract

Considering the limited communication resources and slow convergence speed of multi-unmanned aerial vehicle (UAV) systems, this paper presents a finite-time even-triggered control framework for multi-UAV systems to achieve formation-containment tracking control. First, a virtual leader with time-varying output is introduced so that the trajectory of the whole system can be manipulated in real time. Second, the finite-time control enables that the systematic error converge to a small neighborhood of origin in finite time. Third, in order to save communication resources, an event-triggering function is developed to generate the control event sequences, which avoids continuous update of the controller. Rigorous proof shows the finite-time stability of the proposed control algorithm, and Zeno behavior is strictly excluded for each UAV. Finally, some numerical simulations are given to verify the effectiveness of the proposed controllers.

## Introduction

In recent decades, cooperative control of multi-UAV systems has become a hot research topic due to its wide application of load transportation, localization and and other fields^1–5^. Cooperative control problems mainly includes leaderless consensus^6^, leader-following tracking^7,8^, formation^9,10^ and containment^11,12^. Das et al.^13^ and Fax and Murray^14^ described several methods of formation control, Ge et al.^15^ studied formation tracking control problem based on potential field, took the average position of all agents as virtual leaders, and transformed the task into controlling virtual leaders to track the center of the desired formation. In addition to the formation control problem, containment control also attract the attention of many people.The goal of containment control is to drive all followers to enter the convex hull formed by the leaders^16^. In^17^, the containment control of the multi-agent system under switched topology was discussed. Haghshenas et al.^18^ solved the containment control problem of heterogeneous linear multi-agent systems.

Inspired by the containment control and the formation control, a more complicated formation-containment problem arises, where the leaders need to accomplish a desired formation and the followers are required to converge to the convex hull spanned by the leaders simultaneously. Liu et al.^19^ considered the influence of control input with time delay on formation-containment control of multi-agent system. Han et al.^20^ and Dong et al.^21^ designed the formation-containment control protocol for second-order and high-order multi-agent systems, and used a Riccati equation to solve the gain matrix in the control protocol. In^22^, for the multi-agent system with uncertain nonlinear dynamics and directed communication constraints, a distributed adaptive control approach was proposed to complete the formation-containment target.

It should be pointed out that the macroscopic movement of the whole system cannot be controlled effectively and flexibly in the above formation-containment works^19–22^. In the cooperative transportation application of a group of UAVs crossing dangerous areas, in addition to completing the task of formation-containment, the multi-UAV system should also track the desired trajectory so that all the UAVs can avoid the dangerous area and reach the destination safely. Therefore, the problem of formation-containment tracking appears. In^23,24^, the formation-containment tracking problem was solved for high-order multi-agent system. It can be seen that consensus tracking problem, containment problem and formation problem are special cases of formation-containment tracking problem^23^. How to make the whole system move flexibly and effectively is a challenge to enable it to cope with complex real environments.

It is worth noting that most of the existing research focuses on the asymptotic convergence of multi-agent systems^20,25^. In practical applications, multi-UAV systems require fast response. Compared with the asymptotic convergence algorithm, the finite-time control protocol has faster convergence speed and better anti-interference ability, therefore, it is necessary to study the finite-time control of multi-agent systems. In^9^, the finite-time time-varying formation tracking problem of multi-agent systems was studied in directed topology. A finite-time formation tracking protocol was provided in the presence of mismatched disturbances for high-order multi-agent systems in^26^. Yu et al.^11^ developed a distributed finite-time sliding mode observer for estimating the reference for each UAV, and designed a finite-time containment controller. Nevertheless, these studies only consider formation tracking problem or containment control separately, but do not solve the problem of formation-containment. Namely, It is not guaranteed to complete the formation-containment tracking task in the finite time.

Furthermore, it should be pointed out that the above finite-time controllers employ the continuous time control method, which means that the controller needs to constantly update its control input. However, in practical applications, UAVs are usually equipped with embedded microprocessors with limited computing resources. In view of this concern, event-triggered control is developed to avoid the controller being constantly updated^27–29^. In^1^, aiming at the collaborative transportation problem of the multi-UAV system, a self-triggered method was designed to lower the communication frequency. Chen et al.^30^ discussed the event-triggered formation-containment problem of Euler Lagrange system. In^31^, the formation control problem of the multi-UAV system was considered through a dynamic event-triggered control algorithm, which includes a dynamic threshold. Combining event-triggered control with existing cooperative control is of great value to multi-agent coordinated control problems. The main challenge of event-triggered control is to design triggering functions according to different task scenarios to ensure convergence, and not Zeno behavior occurs.

Motivated by challenges stated above, considering both the limited communication resources and slow convergence speed of the multi-UAV system, this paper investigates the finite-time event-triggered formation-containment tracking control for the multi-UAV system. Compared with the existing literature, the main contributions of this paper are summarized as follows.

- a virtual leader with time-varying output is introduced so that the trajectory of the whole system can be manipulated in real time
- The distributed finite-time protocols are utilized to ensure that the multi-UAV system realize the expected formation-containment tracking in finite time. The upper bound of settling time is given by carefully constructing the Lyapunov function.
- The event-triggering function is developed in the multi-UAV system, which avoids the continuous update input of the controller and greatly reduces the dependence on communication resources.

The remaining sections of this paper are organized as follows. In “Preliminaries and problem formulation”, some basic preliminaries and problem formulation are introduced. “Formation-containment tracking protocol design” presents the designs of formation-containment tracking protocol and analyzes the stability of the system. The results of simulation experiments are provided in “Simulation results”. Finally, the conclusion is drawn in “Conclusions”.

*Notations*: For ${\textbf{x}}=\left[ x_1,x_2,\ldots ,x_n \right] ^T$ and a positive constant $\alpha$, we define ${\mathop {\textrm{sig}}\nolimits } {(\mathbf{{x}})^\alpha } = {\left[ {{\mathop {\textrm{sgn}}} \left( {{x_1}} \right) {{\left| {{x_1}} \right| }^\alpha },{\mathop {\textrm{sgn}}} \left( {{x_2}} \right) {{\left| {{x_2}} \right| }^\alpha }, \ldots ,{\mathop {\textrm{sgn}}} \left( {{x_n}} \right) {{\left| {{x_n}} \right| }^\alpha }} \right] ^T}$, where ${\mathop {\textrm{sgn}}} ( \cdot )$ is the signum function. Let $\left\| \cdot \right\|$ and $\textrm{diag}\left\{ \left. \ldots \right\} \right.$ represent the 2-norm, the block-diagonal matrix. $\otimes$ is the Kronecker product. ${\mathbb {R}} ^n,{\textbf{I}}_n,{\textbf{0}}_n$ denote $n\times 1$ real vectors, $n\times n$ identity matrices and $n\times 1$ zero vectors, respectively. $\lambda _{\min }\left( \cdot \right)$, $\lambda _{\max }\left( \cdot \right)$ represent the maximum and minimum eigenvalues of the square matrix, respectively.

## Preliminaries and problem formulation

### Graph theory and lemmas

A directed graph $G=\left( V,E,A \right)$ is used to represent the information interaction relationship of multi-UAV system. $V=\left\{ \left. 1,2,\ldots ,n \right\} \right.$ is the set of nodes in graph *G*. *E* is the set of edges in the graph. In a directed graph, each edge can only represent one-way information transmission. $(i,j)\in E$ only indicates that nodes *i* can transfer information to nodes *j*, and $\left( i,j \right) \in E\nLeftrightarrow \left( j,i \right) \in E$. $A=\left[ a_{ij} \right] _{n\times n}$ is the weighted adjacency matrix of the graph, if there exists an edge between the node *i* and node *j*, namely, $\left( i,j \right) \in E$, then, $a_{ij}=1$, otherwise $a_{ij}=0$. Define the degree matrix as $D=\textrm{diag}\left[ d_{11},\ldots ,d_{nn} \right]$ with $\sum \nolimits _{j=1}^n{a_{ij}}$ and the Laplacian matrix of *G* is defined as $L=D-W$. A directed graph is said to have a spanning tree if there exists at least one node having a directed path to all the other nodes, and that node is called the root node.

**Figure 1:**
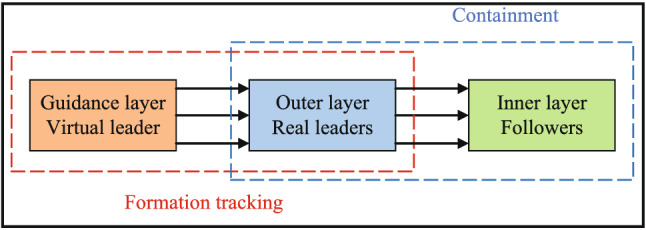
Three-layer structure representation.

The multi-UAV system is represented by three layers, as shown in Fig. 1. The inner layer consists of followers, the real leaders constitute the outer layer, and the guidance layer consists of virtual leader. The inner layer and the outer layer jointly complete the containment task, and the formation tracking target is accomplished by the cooperation of the guidance layer and the outer layer. Assume that the information exchange between two adjacent layers is unidirectional and the information interaction between each agent in the layer is bidirectional. The system consists of $1+N+M$ agents, where $i=0$ represents agent of the guidance layer, namely the virtual leader, $E=\left\{ \left. 1,2,\ldots ,N \right\} \right.$ are the index of the real leaders, which constitute outer layer, and the index set of followers in the inner layer are $F=\left\{ N+1, \right. N+2,\ldots ,\left. N+M \right\}$, then the Laplace matrix $L_A\in {\mathbb {R}} ^{(N+M+1)\times (N+M+1)}$ of the communication topology $G_A$ is expressed in the following form:

$$\begin{aligned} {L_A} = \left[ {\begin{array}{*{20}{l}} 0&{}{{0_{1 \times N}}}&{}{{0_{1 \times M}}}\\ {{L_{12}}}&{}{{L_1}}&{}{{0_{N \times M}}}\\ {{0_{M \times 1}}}&{}{{L_2}}&{}{{L_3}} \end{array}} \right] \end{aligned}$$

where $$L_{12}\in {\mathbb {R}} ^{N\times 1},L_1\in {\mathbb {R}} ^{N\times N},L_2\in {\mathbb {R}} ^{M\times N},L_3\in {\mathbb {R}} ^{M\times M}$$.

Define $B=\textrm{diag}\left\{ \left. L_{12}^{T} \right\} \right.$, and $L=L_1-B$ describes communication topology between real leaders without receiving information from the virtual leader. $L_2$ expresses the communication from real leaders to followers. $L_3$ denotes the communication between followers.

#### Lemma 1

^32^*If exists*, $\delta 1,\delta 2,c>0,p,q>1$ *and* $\frac{1}{p}+\frac{1}{q}=1$, *then* $\delta _1\delta _2\le c^p\frac{\delta _{1}^{p}}{p}+c^{-q}\frac{\delta _{2}^{q}}{q}$.

#### Lemma 2

^33^*For* $x_i\in R$, *if* $\alpha \in [1,+\infty )$, *then* $\left( \sum _{i=1}^n{\left| x_i \right| } \right) ^{\alpha }\ge \sum _{i=1}^n{\left| x_i \right| ^{\alpha }}\ge n^{1-\alpha }\left( \sum _{i=1}^n{\left| x_i \right| } \right) ^{\alpha }$; *and if exists* $\alpha \in \left( 0,1 \right]$, *then* $\left( \sum _{i=1}^n{\left| x_i \right| } \right) ^{\alpha }\le \sum _{i=1}^n{\left| x_i \right| ^{\alpha }}\le n^{1-\alpha }\left( \sum _{i=1}^n{\left| x_i \right| } \right) ^{\alpha }$.

#### Lemma 3

^34^*Consider the system* ${\dot{x}}=f\left( x \right) ,x\in {\mathbb {R}} ^n$, *if there exists a continuous differentiable function* *V*: $[0,\infty )\rightarrow [0,\infty )$, *and it satisfies* ${\dot{V}}(x)\leqslant -c(V(x))^{\eta }$, *where* $c>0$ *and* $0<\eta <1$. *The system can be stabilized in a finite time, and the finite settling time satisfies* $T\le \left( V\left( x_0 \right) \right) ^{1-\eta }/c(1-\eta )$.

#### Assumption 1

The topology between the real leaders and the virtual leader contains a spanning tree, and the root node of the spanning tree is the virtual leader.

#### Assumption 2

The communication topology between followers is a directed graph, and for each follower, there exists at least one directed path from the real leader to the follower.

### Multi-UAV formation-containment tracking problem formulation

For each UAV in the multi-UAV system, the controller design can be divided into inner-loop control and outer-loop control. The inner loop mainly completes the stabilization of the attitude, while the outer loop realizes the tracking of the given trajectory. This paper is mainly concerned with the formation-containment tracking problem of the outer-loop. Define $p_i(t)=\left[ x_i,y_i,z_i \right] ^T$, then the outer-loop dynamic model of each UAV can be described as follows^35^:

1 $$\begin{aligned} \left\{ \begin{array}{l} {\dot{p}}_i=v_i\\ m_i{\dot{v}}_i=-T_{\tau _i}R_ie_3+m_ige_3\,\,i=1,2,\ldots ,n\\ \end{array} \right. \end{aligned}$$

where $p_i\in {\mathbb {R}} ^3,v_i\in {\mathbb {R}} ^3$ and $R_i\in {\mathbb {R}} ^{3\times 3}$ represents the position, velocity and rotation matrix of each UAV, $e_3=[0,0,1]^{\textrm{T}},T_{\tau _i}$ represents the total lift, $m_i,g$ are the mass of the UAV and gravitational acceleration, respectively. Define the control input vector of each UAV $u_i=-\frac{T_{\tau i}}{m_i}R_ie_3+ge_3$, then the dynamic model of the UAV (1) can be rewritten by the following double integrator in^36^,

2 $$\begin{aligned} \left\{ \begin{array}{c} {\dot{p}}_i\left( t \right) =v_i\left( t \right) \\ {\dot{v}}_i\left( t \right) =u_i\left( t \right) \\ \end{array} \right. \end{aligned}$$

**Figure 2:**
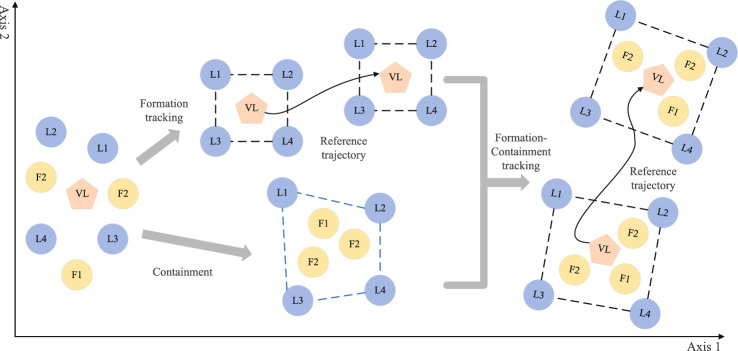
Formation-containment tracking.

Denote $\chi _{i}=\left[ p_{i}^{T},v_{i}^{T} \right] ^T$, $o_i$ represents the position offset vector between the real leaders and the virtual leader.

#### Definition 1

If for all real leaders and any given initial states,

3 $$\begin{aligned} \begin{array}{l} \mathop {\lim }\limits _{t \rightarrow {T_1}} \left\| {{p_i}(t) - {o_i} - {p_0}(t)} \right\| = 0\\ \mathop {\lim }\limits _{t \rightarrow {T_1}} \left\| {{v_i}(t) - {v_0}(t)} \right\| = 0\;\,\;\,\;\,\;\,\;\,\;\,i \in E \end{array} \end{aligned}$$

then in a finite time $T_1$, all the real leaders achieve the expected formation described by $o_i$.

#### Definition 2

For each follower, if there exists $T_2>0$ and non negative constants $\varpi _{ij}$ satisfying $\sum \nolimits _{i=1}^N{\varpi _{ij}}=1$ such that

4 $$\begin{aligned} \lim _{t\rightarrow T_2} \left\| \chi _j(t)-\sum _{i=1}^N{\varpi _{ij}}\chi _i(t) \right\| =0,\forall j\in F,\forall i\in E \end{aligned}$$

then followers is said to achieve containment in a finite time $T_2$.

#### Definition 3

If both the conditions (3) and (4) are established at the same time, namely, a desired formation is formed between real leaders in a finite time $T_1$, the followers converge to the convex hull constructed by the real leaders in a finite time $T_2$, and then Multi-UAV system realizes the finite-time formation-containment tracking, as shown in Fig. 2.

Therefore, the principal problem to be studied in this paper can be expressed as follows: for the UAV system described in (2), design control laws for leaders and followers to achieve the formation-containment tracking goal defined in Definition 3.

## Formation-containment tracking protocol design

### Finite-time event-triggered formation tracking control for leaders

It is worth noting that only real leaders directly connected to the virtual leader can receive virtual leader’s states, therefore, a distributed fixed time observer is designed for each real leader to estimate the state of virtual leader.

5 $$\begin{aligned} \begin{aligned} \ddot{\hat{p}}_i=&\frac{\sum \nolimits _{j=0}^N{a_{ij}}\ddot{\hat{p}}_j}{\sum \nolimits _{j=0}^N{a_{ij}}}-\frac{\alpha }{\sum \nolimits _{j=0}^N{a_{ij}}}{\textrm{sig}}\left( \sum _{j=0}^N{a_{ij}}\left( \hat{\hat{p}}_i-\dot{\hat{p}}_j \right) \right) ^{a/b}\\&-\frac{\beta }{\sum \nolimits _{j=0}^N{a_{ij}}}{\textrm{sig}}\left( \sum _{j=0}^N{a_{ij}}\left( \dot{\hat{p}}_i-\dot{\hat{p}}_j \right) \right) ^{c/d}\\ \end{aligned}i\in E \end{aligned}$$

where $\alpha>0,\beta >0$, *a*, *b*, *c*, *d* are positive odd integers satisfying $a>b,c<d$, $\dot{\hat{p}}_0=v_0$, $\dot{\hat{p}}_i$ represents the estimated velocity of the $i_{th}$ UAV. Under Assumption 1, the observer can estimate the state value in a fixed time^37^.

6 $$\begin{aligned} T<\frac{b}{\alpha (a-b)(3N)^{(1-a/b)/2}}+\frac{d}{\beta (d-c)} \end{aligned}$$

#### Remark 1

When $t\geqslant T$, we can conclude that $\dot{\hat{p}}_1=\dot{\hat{p}}_2=\cdots =\dot{\hat{p}}_N={\dot{p}}_0=v_0$. By further derivation, we get $\ddot{\hat{p}}_1=\ddot{\hat{p}}_2=\cdots =\ddot{\hat{p}}_N=\ddot{p}_0=u_0$.

Describe $\left\{ t_{0}^{i}=0,t_{1}^{i},\ldots ,t_{k}^{i},t_{k+1}^{i},\cdots \right\}$ as the trigger time sequence of $i_{th}$ UAV. The finite-time event-triggered formation tracking controller is designed as follows:

7 $$\begin{aligned} \begin{array}{ll} {u_i} &{}= \ddot{\hat{p}}_{i}\left( {t_k^i} \right) - {k_1}{\mathop {\textrm{sig}}\nolimits } \left[ {\sum \limits _{j = 1}^N {{a_{ij}}} \left( {\left( {{p_i}\left( {t_k^i} \right) - {o_i}} \right) } \right. } \right. \\ &{}\quad -\left. { \left( {{p_j}\left( {t_k^i} \right) - {o_j}} \right) } \right) + {a_{i0}}\left( {{p_i}\left( {t_k^i} \right) - {o_i}} \right) \quad i \in E\\ &{}\quad - {\left. { {p_0}\left( {t_k^i} \right) } \right] ^{\alpha 1}} - {k_2}{\mathop {\textrm{sig}}\nolimits } \left[ {\sum \limits _{j = 1}^N {{a_{ij}}} \left( {{v_i}\left( {t_k^i} \right) - {v_j}\left( {t_k^i} \right) } \right) } \right. \\ &{}\quad - {\left. { {a_{i0}}\left( {{v_i}\left( {t_k^i} \right) - {v_0}\left( {t_k^i} \right) } \right) } \right] ^{\alpha 2}} \end{array} \end{aligned}$$

where $$k_1,k_2>0,0<\alpha _1<1,\alpha _2=2\alpha _1/\left( 1+\alpha _1 \right) $$.

Define the combinational tracking error as:

8 $$\begin{aligned} \begin{aligned} r_i(t)=&\sum _{j=1}^N{a_{ij}}\left( \left( p_i(t)-o_i \right) -\left( p_j(t)-o_j \right) \right) \\&+a_{i0}\left( p_i(t)-o_i-p_0(t) \right) \\ q_i(t)=&\sum _{j=1}^N{a_{ij}}\left( v_i(t)-v_j(t) \right) +a_{i0}\left( v_i(t)-v_0(t) \right) \end{aligned} \end{aligned}$$

Define $R=\left[ r_{1}^{T},\ldots ,r_{N}^{T} \right] ^T$,$Q=\left[ q_{1}^{T},\ldots ,q_{N}^{T} \right] ^T$, where $r_i=\left[ r_{i1},r_{i2},r_{i3} \right] ^T$ ,$q_i=\left[ q_{i1},q_{i2},q_{i3} \right] ^T$.Define the tracking errors as: ${\tilde{p}}_i=p_i(t)-o_i-p_0(t)$,${\tilde{v}}_i=v_i(t)-v_0(t)$. The measurement errors are defined as follows:

9 $$\begin{aligned} \begin{aligned}{}&\varphi _{i}^{r}={\textrm{sig}}\left( r_i\left( t_{k}^{i} \right) \right) ^{\alpha _1}-{\textrm{sig}}\left( r_i(t) \right) ^{\alpha _1}\\&\varphi _{i}^{q}={\textrm{sig}}\left( q_i\left( t_{k}^{i} \right) \right) ^{\alpha _2}-{\textrm{sig}}\left( q_i(t) \right) ^{\alpha _2}\\&\varphi _{i}^{c}=\ddot{\hat{p}}_i\left( t_{k}^{i} \right) -u_0(t)\\\end{aligned} \end{aligned}$$

where $$\varphi _i^r = {\left[ {\varphi _{i1}^r,\varphi _{i2}^r,\varphi _{i3}^r} \right] ^T},\varphi _i^q = {\left[ {\varphi _{i1}^q,\varphi _{i2}^q,\varphi _{i3}^q} \right] ^T},\varphi _i^c = {\left[ {\varphi _{i1}^c,\varphi _{i2}^c,\varphi _{i3}^c} \right] ^T}$$

Define ${\tilde{P}}=\left[ {\tilde{p}}_{1}^{T},\ldots ,{\tilde{p}}_{N}^{T} \right] ^T$,${\tilde{V}}=\left[ {\tilde{v}}_{1}^{T},\ldots ,{\tilde{v}}_{N}^{T} \right] ^T$ where ${\tilde{p}}_i=\left[ {\tilde{p}}_{i1},{\tilde{p}}_{i2},{\tilde{p}}_{i3} \right] ^T$, ${\tilde{v}}_i=\left[ {\tilde{v}}_{i1},{\tilde{v}}_{i2},{\tilde{v}}_{i3} \right] ^T$,then we can get $R=L_1\otimes {\textbf{I}}_3{\tilde{P}}\,\,$and $Q=L_1\otimes {\textbf{I}}_3{\tilde{V}}$, According Remark 1, combine (7), (8) and (9), the system error equation can be described as follows:

10 $$\begin{aligned} \begin{aligned} {\dot{R}}=&Q\\ {\dot{Q}}=&-\left( L_1\otimes {\textbf{I}}_3 \right) \left( k_1{\textrm{sig}}(R)^{\alpha _1}+k_2{\textrm{sig}}(Q)^{\alpha _2} \right. \\&\left. +k_1\varphi ^r+k_2\varphi ^q \right) \\\end{aligned} \end{aligned}$$

where $$\varphi ^r=\left[ \left( \varphi _{1}^{r} \right) ^T,\left( \varphi _{2}^{r} \right) ^T,\ldots ,\left( \varphi _{N}^{r} \right) ^T \right] ^T,\varphi ^q=\left[ \left( \varphi _{1}^{q} \right) ^T \right. ,\left. \left( \varphi _{2}^{q} \right) ^T,\ldots ,\left( \varphi _{N}^{q} \right) ^T \right] ^T.$$

#### Theorem 1

*Let Assumption* 1*hold, then the multi-UAV system* (2) *can achieve the desired formation in finite time under the protocol* (7) *and trigger function* (11). *If exists* $0<\mathrm {\alpha }_1<1,\alpha _2=$ $2\alpha _1/\left( 1+\alpha _1 \right) ,1-3^{\frac{1-\alpha _2}{2}}\xi>0,k_1>k_2(3N)^{\frac{1-\alpha _1}{2\left( 1+\alpha _1 \right) }}(\xi +N)\frac{c^{1+\alpha _1}}{1+\alpha _1},k_1>2^{\frac{2\left( 1+\alpha _1 \right) }{3+\alpha _1}}\left( \sqrt{\frac{2}{\lambda _{\min }\left( L_1 \right) }}\cdot \right.$ $\left. \frac{\theta \lambda _{\max }\left( L_1 \right) \left( 1+\alpha _1 \right) }{3+\alpha _1} \right) ^{1+\alpha _1}\left( 1+ \right.$ $\left. \alpha _1 \right) ^{\frac{1-\alpha _1}{3+\alpha _1}},\frac{k_2\left( 3+\alpha _1 \right) }{2\left( 1+\alpha _1 \right) }\left( 1- \right. \left. 3^{\frac{1-\alpha _2}{2}}\xi \right) \left( \frac{\lambda _{\min }\left( L_1 \right) }{2} \right) ^{\frac{1-\alpha _1}{2\left( 1+\alpha _1 \right) }}>\theta k_2\left( 3N \right) ^{\frac{1-\alpha _1}{2\left( 1+\alpha _1 \right) }}(\xi +N)\frac{\alpha _1c^{-\frac{1+\alpha _1}{\alpha _1}}}{1+\alpha _1}+\theta \lambda _{\max }\left( L_1 \right)$, *where* $c,\theta >0$, *the distributed event-triggered function is designed as follows*:

11 $$\begin{aligned} t_i(t)=\left\| k_1\varphi _{i}^{r}(t) \right\| +\left\| k_2\varphi _{i}^{q}(t) \right\| -\xi \left\| \textrm{sig}\left( q_i(t) \right) ^{\alpha _2} \right\| \end{aligned}$$

*where* $$\xi \in \left( 0,1 \right) $$.

#### Proof

The Lyapunov candidate is constructed as follows :

12 $$\begin{aligned} V_1(t)=k_1\sum _{i=1}^N{\sum _{j=1}^3{\frac{\left| x \right| ^{\alpha _1+1}}{\alpha _1+1}}}(r_{ij})+\frac{1}{2}Q^T\left( {L_1}^{-1}\otimes {\textbf{I}}_3 \right) Q \end{aligned}$$

The derivative of (12) can be obtained:

13 $$\begin{aligned} {{\dot{V}}_1}(t)\mathrm{{ }}&= {k_1}\sum \limits _{i = 1}^N {\sum \limits _{j = 1}^3 {{\mathop {\textrm{sig}}\nolimits } } } {\left( {{r_{ij}}} \right) ^{{\alpha _1}}}{{\dot{r}}_{ij}} + {Q^T}L_1^{ - 1} \otimes {\mathbf{{I}}_3}\dot{Q}\nonumber \\&\quad - {k_2}\sum \limits _{i = 1}^N {q_i^T} {\mathop {\textrm{sig}}\nolimits } {\left( {{q_i}} \right) ^{{\alpha _2}}} - \sum \limits _{i = 1}^N {q_i^T} \left( {{k_1}\varphi _i^r + {k_2}\varphi _i^q} \right) \nonumber \\&\le \sum \limits _{i = 1}^N {\left\| {{q_i}} \right\| } \left( {\left\| {{k_1}\varphi _i^r} \right\| + \left\| {{k_2}\varphi _i^q} \right\| } \right) - {k_2}\sum \limits _{i = 1}^N {q_i^T} {\mathop {\textrm{sig}}\nolimits } {\left( {{q_i}} \right) ^{{\alpha _2}}}\nonumber \\&\le \xi {k_2}\sum \limits _{i = 1}^N {\left\| {{q_i}} \right\| } \left\| {{\mathop {\textrm{sig}}\nolimits } {{\left( {{q_i}} \right) }^{{\alpha _2}}}} \right\| - {k_2}\sum \limits _{i = 1}^N {q_i^T} {\mathop {\textrm{sig}}\nolimits } {\left( {{q_i}} \right) ^{{\alpha _2}}}\nonumber \\&\le - {k_2}\left( {1 - {3^{\frac{{1 - {\alpha _2}}}{2}}}\xi } \right) \sum \limits _{i = 1}^N {{{\left\| {{q_i}} \right\| }^{{\alpha _2} + 1}}} \end{aligned}$$

According to $k_2>0,1-3^{\frac{1-\alpha _2}{2}}\xi >0$, it can be concluded that ${\dot{V}}_1(t)<0$ and the error system (10) is asymptotically stable.

To further prove that the system converges in finite time, the following Lyapunov function is constructed:

14 $$\begin{aligned} V(t)=V_1(t)^{\frac{3+\alpha _1}{2\left( 1+\alpha _1 \right) }}+\theta R^T\left( {L_1}^{-1}\otimes {\textbf{I}}_3 \right) Q \end{aligned}$$

where $\theta$ is a positive constant, according to Lemmas 1 and 2 , the following formula can be obtained

15 $$\begin{aligned}{}&{V_1}{(t)^{\frac{{3 + {\alpha _1}}}{{2\left( {1 + {\alpha _1}} \right) }}}}\nonumber \\&\quad \ge \left( k_1\sum _{i=1}^N{\sum _{j=1}^3{\int _0^{r_{ij}}{\textrm{sig}}}}(x)^{\alpha _1}dx \right) ^{\frac{3+\alpha _1}{2\left( 1+\alpha _1 \right) }}+\left( \frac{1}{2}Q^T\left( {L_1}^{-1}\otimes {\textbf{I}}_3 \right) Q \right) ^{\frac{3+\alpha _1}{2\left( 1+\alpha _1 \right) }} \nonumber \\&\quad \ge {\mathrm{{ }}}{\left( {\frac{{{k_1}}}{{1 + {\alpha _1}}}\sum \limits _{i = 1}^N {\sum \limits _{j = 1}^3 {{{\left| {{r_{ij}}} \right| }^{{\alpha _1} + 1}}} } } \right) ^{\frac{{3 + {\alpha _1}}}{{2\left( {1 + {\alpha _1}} \right) }}}} + {\left( {\frac{{{\lambda _{\min }}\left( {{L_1}} \right) }}{2}\parallel Q\parallel {^2}} \right) ^{\frac{3}{{2\left( {1 + {\alpha _1}} \right) }}}}\nonumber \\&\quad \ge \mathrm{{ }}{\left( {\frac{{{k_1}}}{{1 + {\alpha _1}}}\parallel R\parallel {^{1 + {\alpha _1}}}} \right) ^{\frac{{3 + {\alpha _1}}}{{2\left( {1 + {\alpha _1}} \right) }}}} + {\left( {\frac{{{\lambda _{\min }}\left( {{L_1}} \right) }}{2}\parallel Q\parallel {^2}} \right) ^{\frac{{3 + {\alpha _1}}}{{2\left( {1 + {\alpha _1}} \right) }}}} \end{aligned}$$

16 $$\begin{aligned}{}&\theta {R^T}\left( {{L_1}^{ - 1} \otimes {\mathbf{{I}}_3}} \right) Q\nonumber \\&\quad \ge - \theta {\lambda _{\max }}\left( {{L_1}} \right) \parallel R\parallel \parallel Q\parallel \nonumber \\&\quad \ge - \theta {\lambda _{\max }}\left( {{L_1}} \right) \left( {\frac{2}{{3 + {\alpha _1}}}{h^{\frac{{3 + {\alpha _1}}}{2}}}\parallel R\parallel {^{\frac{{3 + {\alpha _1}}}{2}}} + \frac{{1 + {\alpha _1}}}{{3 + {\alpha _1}}}{h^{ - \frac{{3 + {\alpha _1}}}{{1 + {\alpha _1}}}}}\parallel Q\parallel {^{\frac{{3 + {\alpha _1}}}{{1 + {\alpha _1}}}}}} \right) \end{aligned}$$

where *h* is a positive constant, combining (15) and (16),

17 $$\begin{aligned} {V(t) \ge \left( {{{\left( {\frac{{{k_1}}}{{1 + {\alpha _1}}}} \right) }^{\frac{{3\left( {1 + {\alpha _1}} \right. }}{{2(1 + 1)}}}} - \frac{{2\theta {\lambda _{\max }}\left( {{L_1}} \right) }}{{3 + {\alpha _1}}}{h^{\frac{{3 + {\alpha _1}}}{2}}}} \right) \parallel R\parallel {^{\frac{{3 + {\alpha _1}}}{2}}} }\nonumber \\ +{\left( {\frac{{{\lambda _{\min }}\left( {{L_1}} \right) }}{2} - \frac{{\theta {\lambda _{\max }}\left( {{L_1}} \right) \left( {1 + {\alpha _1}} \right) }}{{3 + {\alpha _1}}}{h^{ - \frac{{3 + {\alpha _1}}}{{1 + {\alpha _1}}}}}} \right) \parallel Q\parallel {^{\frac{{3 + {\alpha _1}}}{{1 + {\alpha _1}}}}}} \end{aligned}$$

In order to ensure that $V\left( t \right)$ is positive definite, satisfying

$$\begin{aligned} {k_1} > {2^{\frac{{2\left( {1 + {\alpha _1}} \right. }}{{3 + {\alpha _1}}}}}{\left( {\sqrt{\frac{2}{{{\lambda _{\min }}\left( {{L_1}} \right) }}} \frac{{\theta {\lambda _{\max }}\left( {{L_1}} \right) \left( {1 + {\alpha _1}} \right) }}{{3 + {\alpha _1}}}} \right) ^{1 + {\alpha _1}}}{\left( {1 + {\alpha _1}} \right) ^{\frac{{1 - {\alpha _1}}}{{3 + {\alpha _1}}}}} \end{aligned}$$

Further, taking the derivative of $V\left( t \right)$

18 $$\begin{aligned} \dot{V}(t)&= \frac{{3 + {\alpha _1}}}{{2\left( {1 + {\alpha _1}} \right) }}{V_1}{(t)^{\frac{{1 - {\alpha _1}}}{{2\left( {1 + {\alpha _1}} \right) }}}}{{\dot{V}}_1}(t) + \theta {Q^T}\left( {L_1^{ - 1} \otimes {\mathbf{{I}}_3}} \right) Q\nonumber \\&\quad + \theta {R^T}\left( {{L_1}^{ - 1} \otimes {\mathbf{{I}}_3}} \right) \dot{Q}\nonumber \\&\le - \frac{{{k_2}\left( {3 + {\alpha _1}} \right) }}{{2\left( {1 + {\alpha _1}} \right) }}\left( {1 - {3^{\frac{{1 - {\alpha _2}}}{2}}}\xi } \right) {\left( {\frac{{{\lambda _{\min }}\left( {{L_1}} \right) }}{2}} \right) ^{\frac{{1 - {\alpha _1}}}{{2\left( {1 + {\alpha _1}} \right) }}}}\parallel Q\parallel {^2}\nonumber \\&\quad + \theta {\lambda _{\max }}\left( {{L_1}} \right) \parallel Q\parallel {^2} - \theta {k_1}\parallel R\parallel {^{{\alpha _1} + 1}}\nonumber \\&\quad + \theta {k_2}{(3N)^{\left. {\frac{{1 - {\alpha _1}}}{{2\left( {1 + {\alpha _1}} \right. }}} \right) }}(\xi + N)\parallel R\parallel \parallel Q\parallel {^{{\alpha _2}}}\nonumber \\&\le - \left( {\theta {k_1} - \theta {k_2}{{(3N)}^{\frac{{1 - {\alpha _1}}}{{2\left( {1 + {\alpha _1}} \right) }}}}(\xi + N)\frac{{{c^{1 + {\alpha _1}}}}}{{1 + {\alpha _1}}}} \right) \parallel R\parallel {^{{\alpha _1} + 1}}\nonumber \\&\quad - \left( {\frac{{{k_2}\left( {3 + {\alpha _1}} \right) }}{{2\left( {1 + {\alpha _1}} \right) }}\left( {1 - {3^{\frac{{1 - {\alpha _2}}}{2}}}\xi } \right) {{\left( {\frac{{{\lambda _{\min }}\left( {{L_1}} \right) }}{2}} \right) }^{\frac{{1 - {\alpha _1}}}{{2\left( {1 + {\alpha _1}} \right) }}}}} \right. \nonumber \\&\quad - \left. {\theta {k_2}{{(3N)}^{\frac{{1 - {\alpha _1}}}{{2\left( {1 + {\alpha _1}} \right. }}}}(\xi + N)\frac{{{\alpha _1}{c^{ - \frac{{1 + {\alpha _1}}}{{{\alpha _1}}}}}}}{{1 + {\alpha _1}}} - \theta {\lambda _{\max }}\left( {{L_1}} \right) } \right) \parallel Q\parallel {^2}\nonumber \\&= - {\omega _1}\parallel R\parallel {^{{\alpha _1} + 1}} - {\omega _2}\parallel Q\parallel {^2}\nonumber \\&\le - \omega \left( {\parallel R\parallel {^{{\alpha _1} + 1}} + \parallel Q\parallel {^2}} \right) \end{aligned}$$

where $c>0$, $\omega =\min \left( \omega _1,\omega _{2} \right)$. From $\omega _1,\omega _2>0$, there exists $\,\,{\dot{V}}\left( t \right) <0$.

Similarly, according to Lemmas 1 and  2, it can be obtained that:

19 $$\begin{aligned}{}&{V_1}{(t)^{\frac{{3 + {\alpha _1}}}{{2\left( {1 + {\alpha _1}} \right) }}}}\nonumber \\&\quad \le {2^{\frac{{1 - {\alpha _1}}}{{2\left( {1 + {\alpha _1}} \right) }}}}{\left( {\frac{{{k_1}{N^{\frac{{1 - {\alpha _1}}}{2}}}}}{{1 + {\alpha _1}}}} \right) ^{\frac{{3 + {\alpha _1}}}{{2\left( {1 + {\alpha _1}} \right) }}}}\parallel R\parallel {^{\frac{{3 + {\alpha _1}}}{2}}}\nonumber \\&\qquad + \frac{1}{2}{\lambda _{\max }}{\left( {{L_1}} \right) ^{\frac{{3 + {\alpha _1}}}{{2\left( {1 + {\alpha _1}} \right) }}}}\parallel Q\parallel {^{\frac{{3 + {\alpha _1}}}{{1 + {\alpha _1}}}}} \end{aligned}$$

and

20 $$\begin{aligned}{}&\theta {R^T}\left( {L_1^{ - 1} \otimes {\mathbf{{I}}_3}} \right) Q\nonumber \\&\quad \le \frac{{2\theta {\lambda _{\max }}\left( {{L_1}} \right) }}{{3 + {\alpha _1}}}{h^{\frac{{3 + {\alpha _1}}}{2}}}\parallel R\parallel {^{\frac{{3 + {\alpha _1}}}{2}}}\nonumber \\&\qquad + \frac{{\left( {1 + {\alpha _1}} \right) \theta {\lambda _{\max }}\left( {{L_1}} \right) }}{{3 + {\alpha _1}}}{h^{ - \frac{{3 + {\alpha _1}}}{{1 + {\alpha _1}}}}}\parallel Q\parallel {^{\frac{{3 + {\alpha _1}}}{{1 + {\alpha _1}}}}} \end{aligned}$$

Combining (19) and (20), we get

21 $$\begin{aligned} V(t)&\le {\beta _1}\parallel R\parallel {^{\frac{{3 + {\alpha _1}}}{2}}} + {\beta _2}\parallel Q\parallel {^{\frac{{3 + {\alpha _1}}}{{1 + {\alpha _1}}}}}\nonumber \\&\le \beta \left( {\parallel R\parallel {^{\frac{{3 + {\alpha _1}}}{2}}} + \parallel Q\parallel {^{\frac{{3 + {\alpha _1}}}{{1 + {\alpha _1}}}}}} \right) \end{aligned}$$

where $\beta =\max \left( \beta _1,\beta _2 \right)$. Combining (18), and further obtain

22 $$\begin{aligned} \dot{V}(t)&\le - \omega \left( {\parallel R\parallel {^{1 + {\alpha _1}}} + \parallel Q\parallel {^2}} \right) \nonumber \\&\le - \omega {\left( {\parallel R\parallel {^{\frac{{3 + {\alpha _1}}}{2}}} + \parallel Q\parallel {^{\frac{{3 + {\alpha _1}}}{{1 + {\alpha _1}}}}}} \right) ^{\frac{{2\left( {1 + {\alpha _1}} \right) }}{{3 + {\alpha _1}}}}}\nonumber \\&\le - \omega {\left( {\frac{{V(t)}}{\beta }} \right) ^{\frac{{2\left( {1 + {\alpha _1}} \right) }}{{3 + {\alpha _1}}}}} \end{aligned}$$

According to Lemma 3, the error system (10) satisfies the condition of finite time stability, namely, *R* and *Q* converge to origin in finite time, with the settling time ${T_1} \le \frac{{{\beta ^{\frac{{2\left( {1 + {\alpha _1}} \right) }}{{3 + {\alpha _1}}}}}\left( {3 + {\alpha _1}} \right) }}{{\omega \left( {1 - {\alpha _1}} \right) }}{V^{\frac{{1 - {\alpha _1}}}{{3 + {\alpha _1}}}}}(0)$.Considering $R=\left( L_1\otimes {\textbf{I}}_3 \right) {\tilde{P}}$,$Q=\left( L_1\otimes {\textbf{I}}_3 \right) {\tilde{V}}$ and $L_1$ is positive definite,we soon have ${\tilde{p}}_i={\tilde{v}}_i=0$ when $t\geqslant T_1$,which means that the desired formation is formed in a finite time $T_1$. This completes the proof. $\square$

#### Theorem 2

*Considering Assumption* 1*holds and the conditions of Theorem* 1*are satisfied, Zeno behavior can be excluded in the control protocol* (7) *and distributed triggering function* (11) *for multi-UAV systems*.

#### Proof

For $t\in \left[ t_{k}^{i},t_{k+1}^{i} \right)$, we derive

23 $$\begin{aligned}{}&\left\| {{k_1}{\dot{\varphi }} _i^r(t)} \right\| + \left\| {{k_2}{\dot{\varphi }} _i^q(t)} \right\| \nonumber \\&\quad = \left\| {{k_1}{\mathop {\textrm{sig}}\nolimits } {{\left( {{{\dot{r}}_i}(t)} \right) }^{{\alpha _1}}}} \right\| + \left\| {{k_2}{\mathop {\textrm{sig}}\nolimits } {{\left( {{{\dot{q}}_i}(t)} \right) }^{{\alpha _2}}}} \right\| \nonumber \\&\quad \le {k_1}{3^{\frac{{1 - {\alpha _1}}}{2}}}{\left\| {{q_i}(t)} \right\| ^{{\alpha _1}}} + {k_2}{3^{\frac{{1 - {\alpha _2}}}{2}}}{\left\| {{{\dot{q}}_i}(t)} \right\| ^{{\alpha _2}}} \end{aligned}$$

when $t=t_{k}^{i}$, $\left\| \varphi _{i}^{r}\left( t_{k_i}^{i} \right) \right\| =0,\left\| \varphi _{i}^{q}\left( t_{k_i}^{i} \right) \right\| =0$, and then

24 $$\begin{aligned}{}&\left\| {{k_1}\varphi _i^r(t)} \right\| + \left\| {{k_2}\varphi _i^q(t)} \right\| \nonumber \\&\quad = \int _{t_k^i}^t {\left( {\left\| {{k_1}{\dot{\varphi }} _i^r(t)} \right\| + \left\| {{k_2}{\dot{\varphi }} _i^q(t)} \right\| } \right) } dt\nonumber \\&\quad \le {k_1}{3^{\frac{{1 - {\alpha _1}}}{2}}}\int _{t_k^i}^t {{{\left\| {{q_i}(t)} \right\| }^{{\alpha _1}}}} dt + {k_2}{3^{\frac{{1 - {\alpha _2}}}{2}}}\int _{t_k^i}^t {{{\left\| {{{\dot{q}}_i}(t)} \right\| }^{{\alpha _2}}}} dt\nonumber \\&\quad \le 2{\delta _1}\int _{t_k^i}^t {{\delta _2}} dt = 2{\delta _1}{\delta _2}\left( {t - t_k^i} \right) \end{aligned}$$

where $\delta _1=\max \left( 3^{\frac{1-\alpha _1}{2}}k_1,3^{\frac{1-\alpha _2}{2}}k_2 \right) ,\delta _2=\max \left( \left\| q_i(t) \right\| ^{\alpha _1} \right. ,\left. \left\| {\dot{q}}_i(t) \right\| ^{\alpha _2} \right)$. When $\Vert q_i\Vert =0$, the formation has been formed, and the controller does not need to be updated. Zeno behavior is naturally excluded. when formation is not achieved, we get $\left\| q_i(t) \right\| ^{\alpha }>0$, According to the event-triggered function (11), we further obtain

25 $$\begin{aligned} 2\delta _1\delta _2\left( t_{k+1}^{i}-t_{k}^{i} \right)&\geqslant \left\| k_1\varphi _{i}^{r}(t) \right\| +\left\| k_2\varphi _{i}^{q}(t) \right\| \nonumber \\&>3^{\frac{1-a_2}{2}}\xi \left\| q_i \right\| ^{\alpha _2}>0 \end{aligned}$$

From (25), we get

26 $$\begin{aligned} t_{k+1}^{i}-t_{k}^{i}>\frac{3^{\frac{1-a_2}{2}}\xi \left\| q_i \right\| ^{\alpha _2}}{2\delta _1\delta _2}>0 \end{aligned}$$

From (26), The inter-event interval has a strict positive lower bound and zeno behavior does not occur in the system. The proof is completed. $\square$

### Finite-time event-triggered containment control for followers

Describe $t_0,t_1,\ldots ,t_k,\ldots$ as the event trigger time series, where $t_k$ denotes the $k_{th}$ triggered time of followers. The finite-time event-triggered containment controller is designed as follows:

27 $$\begin{aligned} u_i(t)&=-k_3\textrm{sig}\left[ \left. \sum _{j=1}^{N+M}{a_{ij}\left( p_i\left( t_{k}^{i} \right) -p_j\left( t_{k}^{i} \right) \right) } \right] ^{\alpha _3} \right. \nonumber \\&\quad -k_4\textrm{sig}\left[ \sum _{j=1}^{N+M}{a_{ij}\left( v_i\left( t_{k}^{i} \right) -v_j\left( t_{k}^{i} \right) \right) } \right] ^{\alpha _4}\,\, i\in F \end{aligned}$$

where $$k_3,k_4>0$$, $$\alpha _3\in \left( 0,1 \right) $$, $$\alpha _4=\frac{2\alpha _3}{1+2\alpha _3}$$.

The combinational tracking errors are defined as

28 $$\begin{aligned}{}&{\mathcal {E}} _{pi}(t)=\sum _{j=1}^{N+M}{a_{ij}}\left( p_i(t)-p_j(t) \right) \nonumber \\&{\mathcal {E}} _{vi}(t)=\sum _{j=1}^{N+M}{a_{ij}}\left( v_i(t)-v_j(t) \right) \,\, i\in F \end{aligned}$$

Define ${\mathcal {E}} _p=\left[ {\mathcal {E}} _{p1}^{T},\ldots ,{\mathcal {E}} _{pM}^{T} \right] ^T,{\mathcal {E}} _v=\left[ \left. {\mathcal {E}} _{v1}^{T},\ldots ,{\mathcal {E}} _{vM}^{T} \right] ^T \right.$, where ${\mathcal {E}} _{pi}=\left[ {\mathcal {E}} _{pi1},\ldots , \right.$ $\left. {\mathcal {E}} _{pi3} \right] ^T,{\mathcal {E}} _{vi}=\left[ {\mathcal {E}} _{v1},\ldots ,{\mathcal {E}} _{v3} \right] ^T$. Define the tracking errors as

29 $$\begin{aligned} \left\{ \begin{array}{l} {\tilde{\varepsilon }}_{pi}=p_i(t)-p_{\tau }(t)\\ {\tilde{\varepsilon }}_{vi}=v_i(t)-v_{\tau }(t) \end{array} \right. \end{aligned}$$

where $\tau \in E$ being the real leader directly connected to the followers. Define $\tilde{{\mathcal {E}}}_p=\left[ {\tilde{\varepsilon }}_{p1}^{T},\ldots ,{\tilde{\varepsilon }}_{pM}^{T} \right] ^T$, $\tilde{{\mathcal {E}}}_v=\left[ {\tilde{\varepsilon }}_{v1}^{T}, \right. \ldots ,\left. {\tilde{\varepsilon }}_{vM}^{T} \right] ^T$,where ${\tilde{\varepsilon }}_{pi}=\left[ {\tilde{\varepsilon }}_{pi1},{\tilde{\varepsilon }}_{pi2},{\tilde{\varepsilon }}_{pi3} \right] ^T,{\tilde{\varepsilon }}_{vi}=\left[ {\tilde{\varepsilon }}_{vi1},{\tilde{\varepsilon }}_{vi2},{\tilde{\varepsilon }}_{vi3} \right] ^T$ then we get ${\mathcal {E}} _p=L_3\otimes {\textbf{I}}_3\tilde{{\mathcal {E}}}_p,{\mathcal {E}} _v=L_3\otimes {\textbf{I}}_3\tilde{{\mathcal {E}}}_v$. Define the measurement errors as

30 $$\begin{aligned} \mathrm {\zeta }_{i}^{p}&=\textrm{sig}\left( {\mathcal {E}} _{pi}\left( t_{k}^{i} \right) \right) ^{\alpha _3}-\textrm{sig}\left( {\mathcal {E}} _{pi}(t) \right) ^{\alpha _3}\nonumber \\ \mathrm {\zeta }_{i}^{v}&=\textrm{sig}\left( {\mathcal {E}} _{vi}\left( t_{k}^{i} \right) \right) ^{\alpha _4}-\textrm{sig}\left( {\mathcal {E}} _{vi}(t) \right) ^{\alpha _4} \end{aligned}$$

where $\zeta _{i}^{p}=\left[ \zeta _{i1}^{p},\zeta _{i2}^{p},\zeta _{i3}^{p} \right] ^T,\zeta _{i}^{v}=\left[ \zeta _{i1}^{v},\zeta _{i2}^{v},\zeta _{i3}^{v} \right] ^T$. Combining (27), (28) and (30), the dynamic model of the Multi-UAV system can be rewritten as

31 $$\begin{aligned} \dot{{\mathcal {E}}}_p&= {{{{\mathcal {E}}}}_v}\nonumber \\ \dot{{\mathcal {E}}}_v&= - \left( {{L_3} \otimes {\mathbf{{I}}_3}} \right) \left( {{k_3}{\mathop {\textrm{sig}}\nolimits } {{\left( {{{{{\mathcal {E}}}}_p}} \right) }^{{\alpha _3}}} + {k_4}{\mathop {\textrm{sig}}\nolimits } {{\left( {{{{{\mathcal {E}}}}_v}} \right) }^{{\alpha _4}}}} \right. \nonumber \\&\quad \left. { + {k_3}{\zeta ^p} + {k_4}{\zeta ^v}} \right) \end{aligned}$$

where $$\zeta ^p=\left[ \left( \zeta _{1}^{p} \right) ^T,\left( \zeta _{2}^{p} \right) ^T,\ldots ,\left( \zeta _{M}^{p} \right) ^T \right] ^T,\zeta ^v=\left[ \left( \zeta _{1}^{v} \right) ^T, \right. \left. \left( \zeta _{2}^{v} \right) ^T,\ldots ,\left( \zeta _{M}^{v} \right) ^T \right] ^T$$.

Moreover, The triggering function for the $i_{th}$ follower is designed as

32 $$\begin{aligned} t_i(t)=\left\| k_3\zeta _{i}^{p}(t) \right\| +\left\| k_4\zeta _{i}^{v}(t) \right\| -\eta \left\| \textrm{sig}\left( {\mathcal {E}} _{vi}(t) \right) ^{\alpha _4} \right\| \end{aligned}$$

where $\eta \in \left( 0,1 \right)$ is the parameter that can be adjusted. The triggering condition is defined as $t_{k+1}^{i}=\textrm{inf}\left\{ t>t_{k}^{i}, \right. t_i\left. (t)>0 \right\}$.

#### Theorem 3

*When the Assumption* 2*holds, followers use control protocol* (27) *and distributed triggering function* (32), *if exists* $\vartheta ,\kappa >0$, *conditions in Theorem*1*hold and* $k_3$, $k_4$ *satisfy the inequalities* (33), (34), *all followers can converge to the convex hull spanned by the real leaders in a finite time. Moreover the Zeno behavior of the containment system is excluded*.

33 $$\begin{aligned}{}&k_3>2^{\frac{2\left( 1+\alpha _3 \right) }{3+\alpha _3}}\left( \sqrt{\frac{2}{\lambda _{\min }\left( L_3 \right) }}\frac{\vartheta \lambda _{\max }\left( L_3 \right) \left( 1+\alpha _3 \right) }{3+\alpha _3} \right) ^{1+\alpha _3}\left( 1+\alpha _3 \right) ^{\frac{1-\alpha _3}{3+\alpha _3}} \end{aligned}$$

34 $$\begin{aligned}{}&\frac{k_4\left( 3+\alpha _3 \right) }{2\left( 1+\alpha _3 \right) }\left( 1-3^{\frac{1-\alpha _2}{2}}\eta \right) \left( \frac{\lambda _{\min }\left( L_3 \right) }{2} \right) ^{\frac{1-\alpha _3}{2\left( 1+\alpha _3 \right) }} \nonumber \\&\quad >\vartheta k_4\,\,(3M)^{\frac{1-\alpha _3}{2\left( 1+\alpha _3 \right) }}\left( \eta + \right. \left. M \right) \frac{\alpha _3\kappa ^{-\frac{1+\alpha _3}{\alpha _3}}}{1+\alpha _3}+\vartheta \lambda _{\max }\left( L_3 \right) \end{aligned}$$

#### Proof

From Theorem 1 and the previous proof, we take the same procedure of the proof for followers, one can prove that under the control protocol (27), ${\mathcal {E}} _p$ and ${\mathcal {E}} _v$ can converge to the origin in finite time $T_2$. Further to exclude the Zeno behavior, by employing the same procedure of the proof for real leaders in Theorem 2, we can conclude that the inter-event interval has a positive lower bound.

35 $$\begin{aligned} \varDelta >\frac{3^{\frac{1-a_4}{2}}\eta \left\| {\mathcal {E}} _{vi} \right\| ^{\alpha _4}}{2\mu _1\mu _2} \end{aligned}$$

where $\varDelta$ denotes the inter-event interval, $\mu _1=\max \left( 3^{\frac{1-\alpha _3}{2}}k_3,3^{\frac{1-\alpha _4}{2}}k_4 \right) ,\mu _2=$ $\max \left( \left\| {\mathcal {E}} _{vi}(t) \right\| ^{\alpha _3},\left\| \dot{{\mathcal {E}}}_{vi}(t) \right\| ^{\alpha _4} \right)$. This completes the proof. $\square$

#### Remark 2

The conclusion of Theorem 3 is established on the basis of Theorem 1. when the real leaders achieve the desired formation, the followers can enter the convex hull generated by the the real leaders and the multi-UAV system (2) eventually accomplish the formation-containment tracking task.

**Figure 3:**
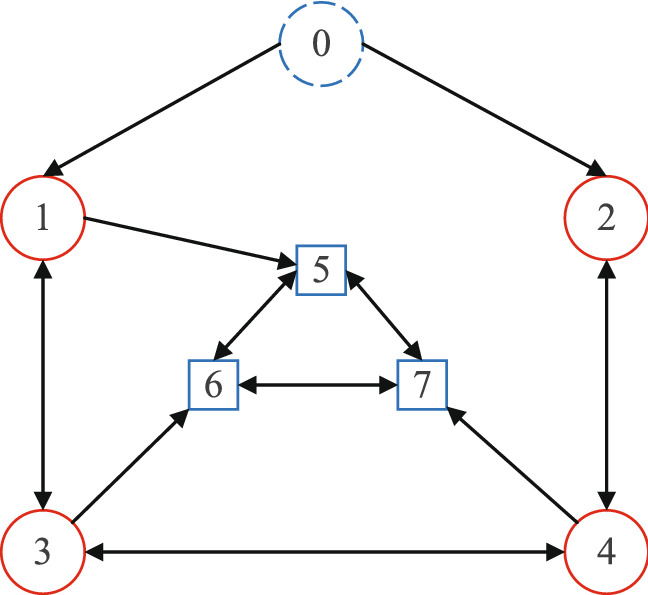
The interaction topology among 7 UAVs.

## Simulation results

In order to verify the validity and superiority of the proposed method in achieving formation containant tracking, this section uses a scenario containing 7 UAVs to conduct a simulation experiment. The People Computer(PC) is equipped with Intel(R) Core(TM) i5-8300H 2.3GHZ CPU, 8GB memory. Simulations are executed in MATLAB environment.The UAVs in the Multi-UAV system are depicted by the dynamic model (2) with $p_i=\left[ p_{i1},p_{i2},p_{i3} \right] ^T,v_i=\left[ v_{i1},v_{i2},v_{i3} \right] ^T,i=0,1,\ldots ,7,$ where $p_{i1,}p_{i2}$ and $p_{i3}$ describe the positions in the directions X, Y and Z, $v_{i1,}v_{i2}$ and $v_{i3}$ are the velocities in X, Y and Z. The Multi-UAV system consists of the guidance UAV denoted by $i=0$, the outer UAVs represented by index set $E=\left\{ 1,2,3,4 \right\}$ and the inner UAVs represented by index set $F=\left\{ 5,6,7 \right\}$. The information interaction topology between UAVs is shown in Fig. 3. The initial state of each agent in the Multi-UAV system is selected as follows: $p_{ij}(0)=10\varTheta \left( i=1, \right. 2,3,4,5,6,7;j=1,2,$ $\left. 3 \right) ,v_{ij}\left( 0 \right) =\varTheta \left( i=1,2,3,4, \right. 5,6,7;\left. j=1,2,3 \right)$ , where $\varTheta$ is a random number in the range $\left( 0,1 \right)$. The guidance and outer UAVs require to accomplish a formation tracking with a square configuration, where the offset vectors are shown as $o_1=[0,-10,0]^T$, $o_2=[-10,0,0]^T$, $o_3=[0,10,0]^T$, $o_4=[10,0,0]^T$.

**Figure 4:**
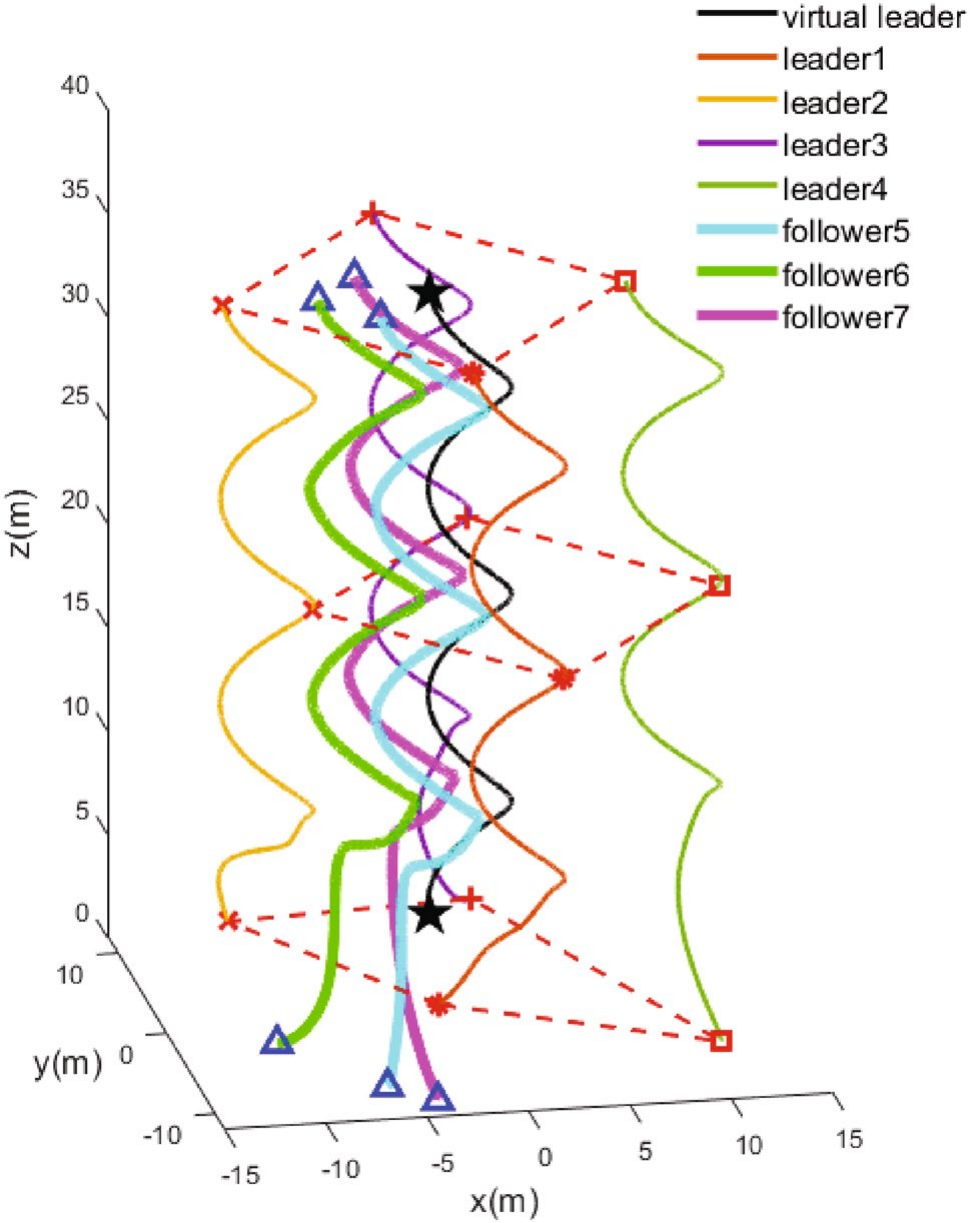
Three dimensional trajectory of UAVs.

**Figure 5:**
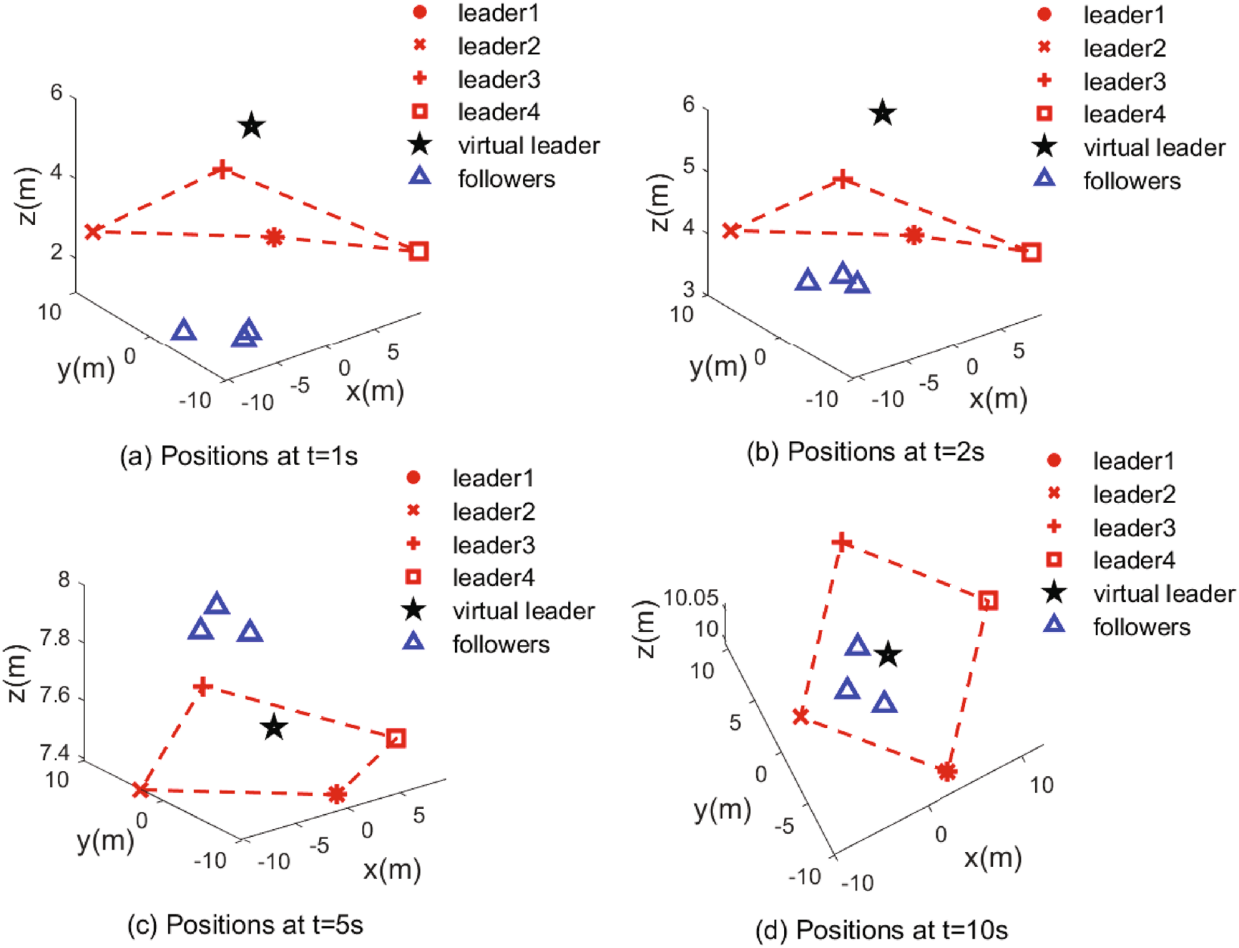
Position snapshots at t = 1 s, 2 s, 5 s, 10 s for the UAVs.

**Figure 6:**
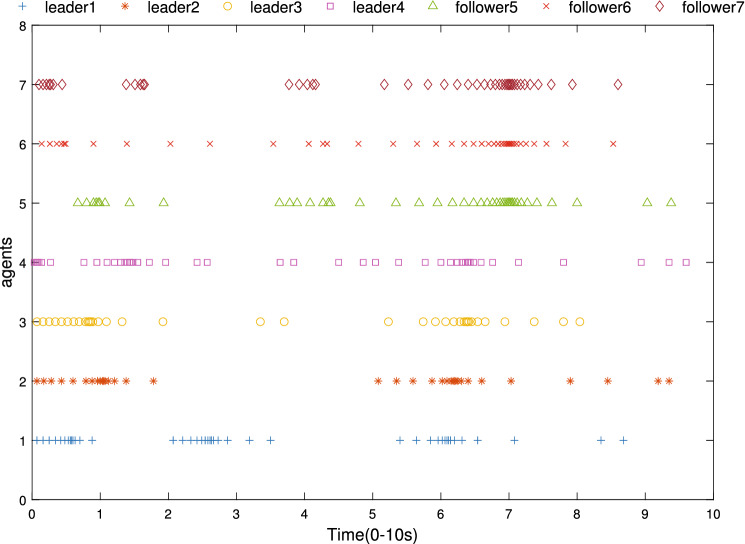
Events triggering times of UAVs.

**Figure 7:**
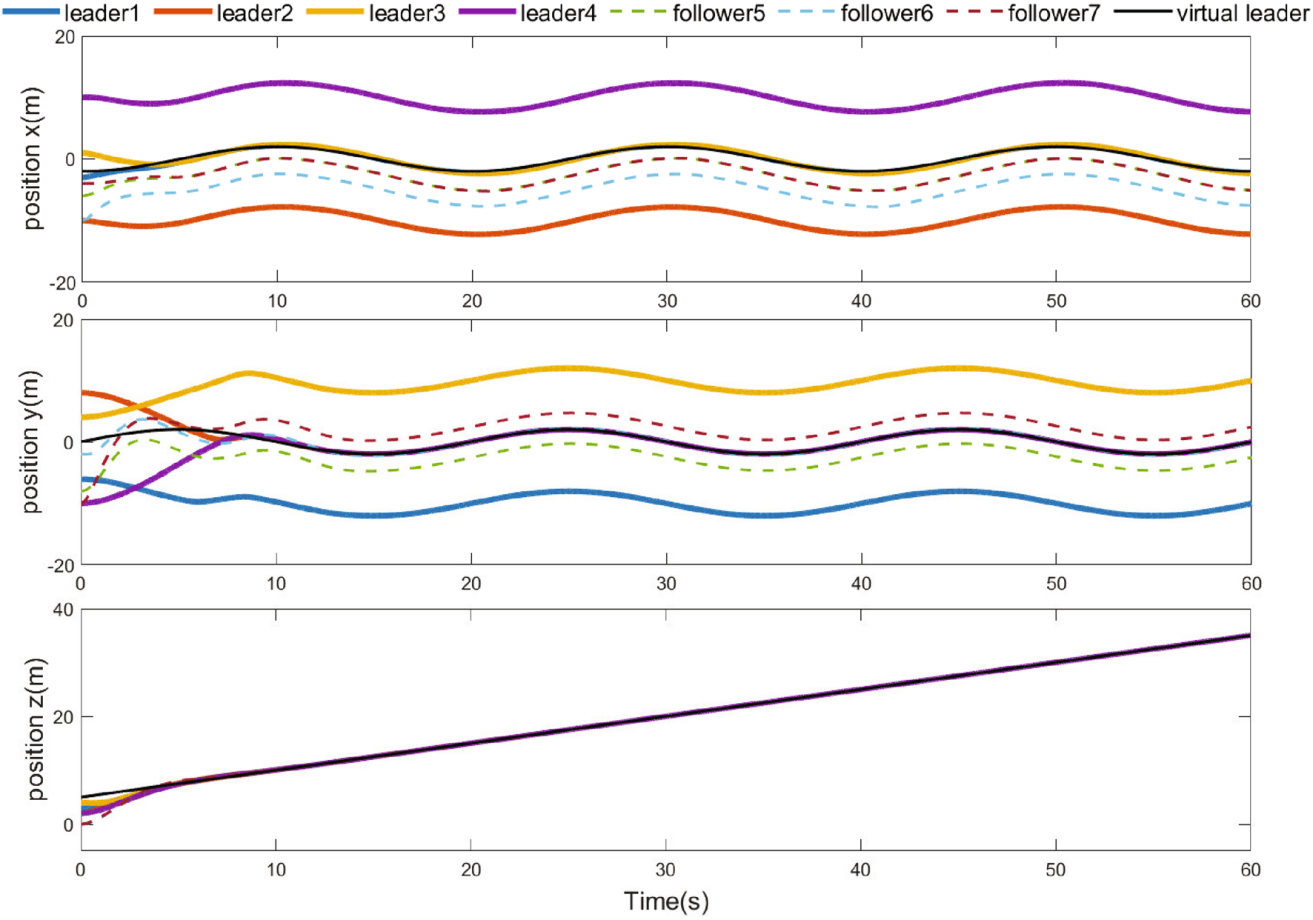
Positions of UAVs.

**Figure 8:**
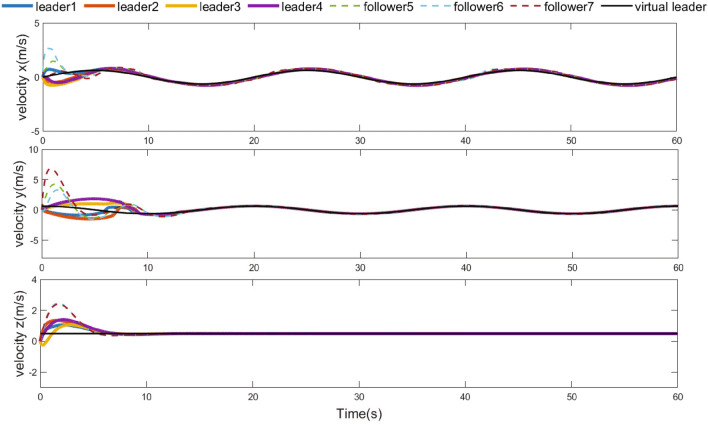
Velocities of UAVs.

The guidance UAV leads the multi-UAV system with the following desired time-varying reference trajectory:

36 $$\begin{aligned} {\left\{ \begin{array}{ll} p_{01}=-2\cos \left( 0.1\mathrm {\pi }t \right) +1\\ p_{02}=2\sin \left( 0.1\mathrm {\pi }t \right) \\ p_{03}=0.5t+2\\ \end{array}\right. } \end{aligned}$$

The coefficients of observer (5) are chosen as $\alpha =\beta =5,a=d=7,b=c=5$ and the control parameters of protocols (7) and (27) are selected as $k_1=4,k_2=2,\alpha _1=0.7,\alpha _2=$ $\frac{2\alpha _1}{1+\alpha _1},k_3=6,k_4=3,\alpha _3=0.8,\alpha _4=\frac{2\alpha _3}{1+\alpha _3},$ Choose $\xi =0.5,\eta =0.8$ as the parameters of the triggering functions (11) and (32). All parameters are the same on all three channels.

**Figure 9:**
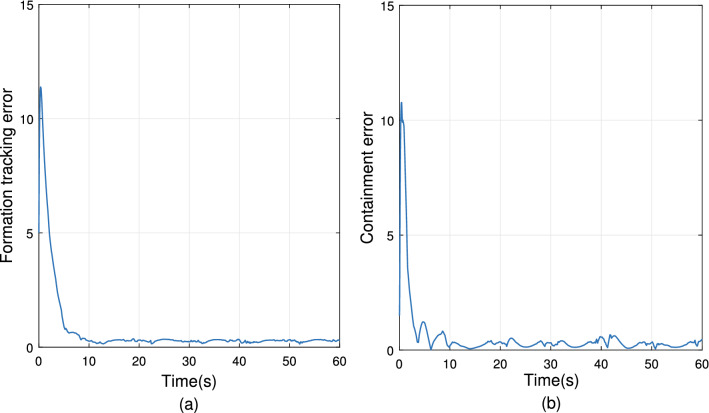
Curves of the formation tracking error for the leaders and the containment error for the followers. (**a**) Formation tracking error. (**b**) Containment error.

The simulation results are demonstrated from Figs. 4, 5, 6, 7, 8 and 9. Figure 4 shows the three-dimensional trajectory of the UAVs. Figure 5 shows the position snapshots of the multi-UAV system at different times *t*=1 s, 2 s, 5 s and 10 s. Figure 6 gives the events triggering times of 7 UAVs within 10 s. The evolutions of the position and velocity of each UAV in the X, Y and Z directions are given in Figs. 7 and  8. The formation tracking error displayed in Fig. 9a is defined as $\parallel \varGamma _E\parallel =\parallel \varGamma _E\parallel _1$, with $\varGamma _E=\left[ \varGamma _{E1},\varGamma _{E2},\varGamma _{E3},\varGamma _{E4} \right] ^T$, where $\forall i=1,2,3,4,h_i=\left[ o_i,0_3 \right] ^T,\left\| \varGamma _i \right\| =\left\| \chi _i-h_i- \right. \left. -\chi _0 \right\| _1$. Similarly, the containment error $\varGamma _C$ is obtained and shown in Fig. 9b.

From Figs. 4, 5, 6, 7, 8 and 9, it can be clearly seen that the outer UAVs achieve the desired formation in a finite time, the inter UAVs enter the convex hull, and the task of formation-containment tracking is accomplished. In addition, with the time-varying of the state of guidance UAV, the multi-UAV system can still realize formation-containment tracking, which shows the robustness of the method proposed in this paper. At the initial time *t*= 0, the UAVs are randomly distributed in the space. After 1 s, in order to realize the formation, the outer UAVs start to move. At *t*= 2 s, the inner UAVs converge to the convex hull, and at *t*= 5 s and at 10 s of the snapshots show that the outer UAVs slowly achieve the square formation around the guidance UAV, and the inner UAVs enter the convex hull formed by the outer UAVs. Figure 6 shows that Zeno behavior does not occur, and the minimum interval of two adjacent events in the leaders’ system and followers’ system are 0.2 s and 0.4 s respectively, which indicates that the internal communication of the system is intermittent, not continuously. We can also see from Fig. 9 that the systematic error of the UAVs converges to the origin within 10 s. Therefore, under protocols (7) and (27), and with the triggering function satisfying (11) and (32), the multi-UAV system can realize the finite-time event-triggered formation-containment tracking.

## Conclusions

In this paper, the problem of time-varying out formation-containment tracking of multi-UAV systems is studied, with a three-layer hierarchical structure being introduced. A finite-time event-triggered control protocol is proposed for real leaders and followers, respectively. The problems of limited communication resources and slow convergence speed in multi-UAV systems are solved at the same time. The simulation results show that the time-varying tracking task can be completed in finite time, and the system does not exist zeno behavior. In future work, we will consider the obstacle avoidance problem in the tracking process, and consider the output saturation of the system to make the system more robust.

## Data Availability

All data included in this study are available upon request by contact with the corresponding author.
